# Ibrutinib synergizes with MDM-2 inhibitors in promoting cytotoxicity in B chronic lymphocytic leukemia

**DOI:** 10.18632/oncotarget.12139

**Published:** 2016-09-20

**Authors:** Rebecca Voltan, Erika Rimondi, Elisabetta Melloni, Gian Matteo Rigolin, Fabio Casciano, Maria Vittoria Arcidiacono, Claudio Celeghini, Antonio Cuneo, Giorgio Zauli, Paola Secchiero

**Affiliations:** ^1^ Department of Morphology, Surgery and Experimental Medicine and LTTA Centre, University of Ferrara, Ferrara, Italy; ^2^ Department of Life Sciences, University of Trieste, Trieste, Italy; ^3^ Department of Medical Sciences, Section of Hematology, University of Ferrara, Ferrara, Italy

**Keywords:** B leukemic cells, Ibrutinib, MDM-2 inhibitors, apoptosis, combination therapy

## Abstract

**Objective:**

The aim of this study was to investigate the anti-leukemic activity of the Bruton tyrosine kinase inhibitor Ibrutinib in combination with the small molecule MDM-2 inhibitor Nutlin-3 in preclinical models.

**Methods:**

The potential efficacy of the Ibrutinib/Nutlin-3 combination was evaluated *in vitro* in a panel of B leukemic cell lines (EHEB, JVM-2, JVM-3, MEC-1, MEC-2) and in primary B-chronic lymphocytic leukemia (B-CLL) patient samples, by assessing cell viability, cell cycle profile, apoptosis and intracellular pathway modulations. Validation of the combination therapy was assessed in a B leukemic xenograft mouse model.

**Results:**

Ibrutinib exhibited variable anti-leukemic activity *in vitro* and the combination with Nutlin-3 synergistically enhanced the induction of apoptosis independently from the p53 status. Indeed, the Ibrutinib/Nutlin-3 combination was effective in promoting cytotoxicity also in primary B-CLL samples carrying 17p13 deletion and/or *TP53* mutations, already in therapy with Ibrutinib. Molecular analyses performed on both B-leukemic cell lines as well as on primary B-CLL samples, while confirming the switch-off of the MAPK and PI3K pro-survival pathways by Ibrutinib, indicated that the synergism of action with Nutlin-3 was independent by p53 pathway and was accompanied by the activation of the DNA damage cascade signaling through the phosphorylation of the histone protein H2A.X. This observation was confirmed also in the JVM-2 B leukemic xenograft mouse model.

**Conclusions:**

Taken together, our data emphasize that the Ibrutinib/Nutlin-3 combination merits to be further evaluated as a therapeutic option for B-CLL.

## INTRODUCTION

Bruton tyrosine kinase (BTK), a nonreceptor tyrosine kinase member of the Tec kinase family, plays a significant role in B-cell development. BTK is a key component of the B cell receptor (BCR) signaling that regulates B cell proliferation and survival and is involved in signaling pathways downstream of other receptors [1]. BTK is also known to be important for B cell migration and homing and is activated upon chemokine binding to CXCR4 and CXCR5 through direct interaction with the chemokine receptor G protein subunits [2, 3]. For all these reasons, BTK represents an unique therapeutic target in B-cell malignancies [1]. Inhibition of BTK in B chronic lymphocytic leukemia (B-CLL) disrupts integrin-mediated adhesion to fibronectin, diminishes cellular response to tissue homing chemokines, counteracts NFκB DNA binding, inhibits DNA synthesis and induces moderate apoptosis, thus affecting cell survival, proliferation and migration [4–6].

Among BTK inhibitors actually in preclinical development and clinical trials, Ibrutinib is the most advanced molecule for the treatment of B-cell malignancies [1]. Ibrutinib is an orally available, selective and irreversible inhibitor of BTK that covalently binds to Cys481 [7]. In particular, BTK inhibition by Ibrutinib clearly has significant activity across all subtypes of CLL including patients with poor prognostic risk disease [8]. Ibrutinib, as single agent, has been used in elderly patients (≥65 years) for treatment of naïve B-CLL as well as for those with relapsed and refractory disease [8, 9]. An unanticipated finding was that treatment with Ibrutinib induced a prompt lymphocytosis in the peripheral blood. In particular, in Ibrutinib-treated CLL patients the lymphocytosis is usually seen by 7 days, peaking within 4 weeks and then slowly decreasing with time. In contrast, however, lymph node size diminishes rapidly in CLL, with the majority of effects seen in the first 2 months of therapy [9]. The present indication is that there are very few complete responses with Ibrutinib (2% in previously treated CLL, 13% in treatment of naïve CLL) and when treatment is interrupted disease progression is rapidly seen. Therefore, it is intensely debated the search of combination therapy to improve responses, with the aim of attaining a minimal residual disease (MRD) negative response, without significant toxicity for the patients. Moreover, another evidence suggesting the importance of searching new therapeutic combinations with Ibrutinib is the emergence of resistance to Ibrutinib monotherapy [10–13].

In this respect, we and other investigators have demonstrated the potential efficacy of MDM-2 inhibitors, used either alone or in combinations, as anti-leukemic agents [14–18]. Interestingly, a number of studies have shown the potential p53-independent synergism of Nutlin-3 with different anti-cancer drugs [16, 17, 19–21]. On these bases, the aim of this study was to investigate the potential anti-leukemic activity of Ibrutinib/Nutlin-3 combination in preclinical models consisting of a panel of p53^wild-type^ and p53^mutated^ B leukemic cell lines and primary B-CLL patient samples as well as a B leukemic xenograft mouse model.

## RESULTS

### Treatment with Ibrutinib/Nutlin-3 combination exhibits a synergistic anti-leukemic activity in both p53^wild-type^and p53^mutated^ B leukemic cells

In the first group of experiments, Ibrutinib was tested on a panel of p53^wild-type^ (EHEB, JVM-2, JVM-3) and p53^mutated^ (MEC-1, MEC-2) B lymphoblastoid leukemic cell lines. As documented by the IC_50_ values, Ibrutinib showed a variable cytotoxicity independently from the p53 status of the cell line (range 14.8-19 μM and 5.4-14.8 μM, respectively at 24 and 48 hours; Table 1). Analysis of the cytotoxicity induced by treatment with Ibrutinib was then evaluated on primary cells derived from a cohort of B-CLL patients (n=22; Table 2). Analysis of IC_50_ mean values indicated that also B-CLL patient cell samples were susceptible to Ibrutinib independently from the p53 status (Table 2). On the other hand, treatment with Ibrutinib (used at concentrations up to 10 μM) of PBMC isolated from healthy blood donors (n=5) did not significantly affect cell viability (data not shown).

**Table 1:** IC_50_ for Ibrutinib in leukemic B-cell lines

Leukemic cells	IC_50_ Ibrutinib (μM)	
	24 hours	48 hours
EHEB	18	11.5
JVM-2	14.8	5.4
JVM-3	19	14.8
MEC-1	18.6	10
MEC-2	15.3	10

**Table 2:** Clinical and laboratory characteristics of the B-CLL patients

Pt.#	Therapy	%CD38^+^ cells^†^	%ZAP-70^+^	Cytogenetic abnormalities^*^	IgVH status	TP53 status	IC_50_ Ibrutinib (μM)
1	Untreated	neg	neg	del13q	mut	wild-type	14.6
2	Ibrutinib	neg	neg	del11q, del13q	unmut	mut^a^	11
3	Untreated	neg	neg	trisomy 12	mut	wild-type	0.1
4	Untreated	neg	neg	neg	mut	wild-type	14.4
5	Untreated	neg	neg	neg	mut	wild-type	12.3
6	Ibrutinib	pos	na	del11q, del13q	unmut	mut^b^	17.7
7	Untreated	pos	pos	del13q	unmut	wild-type	10.2
8	Untreated	neg	neg	del13q	mut	wild-type	12.5
9	R-Benda	pos	neg	del13q	unmut	wild-type	11.2
10	Untreated	neg	neg	na	mut	wild-type	5.3
11	Untreated	pos	neg	del13q	mut	wild-type	11.8
12	Ibrutinib	neg	neg	del11q, del17p, del13q	unmut	mut^c^	4.4
13	Chl	pos	neg	neg	unmut	wild-type	3.1
14	Untreated	neg	na	neg	na	wild-type	35.9
15	Untreated	neg	neg	del13q	mut	wild-type	5.2
16	R-Benda	neg	neg	del13q	mut	wild-type	2.5
17	FC, FCR, R-Benda	neg	neg	del11q, del13q	na	wild-type	8.1
18	Untreated	pos	na	trisomy 12	mut	wild-type	10.5
19	Untreated	pos	na	trisomy 12	na	na	7.1
20	Untreated	neg	neg	neg	mut	wild-type	2.9
21	Untreated	neg	na	del13q	na	na	15
22	Ibrutinib	neg	na	del13q	unmut	wild-type	17.6

Pt., patient; R-Benda, Rituximab-Bendamustine; Chl, Chlorambucil; FC, Fludarabine-Chlorambucil; FCR, Fludarabine-Chlorambucil-Rituximab; neg, negative; pos, positive; na, not available; mut, mutated; unmut, unmutated.

† Results obtained in cytometric assays, using a CD38 cutoff of 30%.

* Cytogenetic abnormalities were evaluated by fluorescence in situ hybridization (FISH) analysis.

a c.394A>G:p.K132E;

b c.644G>A:p.S215N;

c c.770T>C:p.L257P.

**Figure 1:**
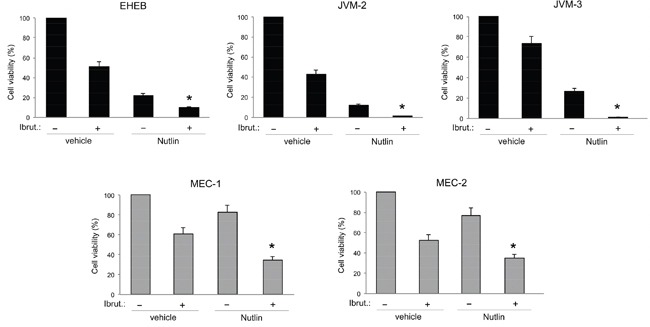
Evaluation of cytotoxic effect in response to Ibrutinib and Nutlin-3 used alone or in combination in B leukemic cell lines The p53^wild-type^ (EHEB, JVM-2, JVM-3) and the p53^mutated^ (MEC-1, MEC-2) leukemic cells were exposed to Ibrutinib (10 μM) and Nutlin-3 (10 μM) used either alone or in combination for 48 hours. Cell viability was calculated as percentage with respect to the control untreated cultures (set to 100% for each cell line). Data are reported as the mean±SD of results from at least three independent experiments. The asterisk indicates p<0.05 respect to single treatments.

On these bases, in order to enhance the anti-leukemic activity of Ibrutinib, we have then assessed the *in vitro* effect of Ibrutinib used in combination with Nutlin-3. As shown in Figure 1, the combined treatment induced a reduction of cell viability significantly higher with respect to the single drugs in all B leukemic cell lines analyzed. Moreover, experiments performed treating cells with serial concentrations of Ibrutinib and Nutlin-3 at constant ratio, and analyzed with the method of Chou and Talalay [22], revealed that the Ibrutinib/Nutlin-3 combination promoted a synergistic (average CI<1) cytotoxicity both in p53^wild-type^ (EHEB, JVM-2, JVM-3) and in p53^mutated^ (MEC-1, MEC-2) B lymphoblastoid leukemic cell lines (Table 3).

**Table 3:** Combination index values for the effect of Ibrutinib/Nutlin-3 combination on viability of leukemic B-cell lines

Leukemic cells	ED50	ED75	ED90	Average CI
EHEB	0.80	0.60	0.48	0.63
JVM-2	0.52	0.50	0.50	0.51
JVM-3	0.49	0.19	0.07	0.25
MEC-1	0.42	0.61	0.88	0.64
MEC-2	0.45	0.54	0.67	0.83

ED indicates effect dose. The average combination index (CI) values were calculated from ED50, ED75 and ED90.

### The synergistic anti-leukemic activity of the Ibrutinib/Nutlin-3 combination is mainly due to induction of apoptosis

**Figure 2:**
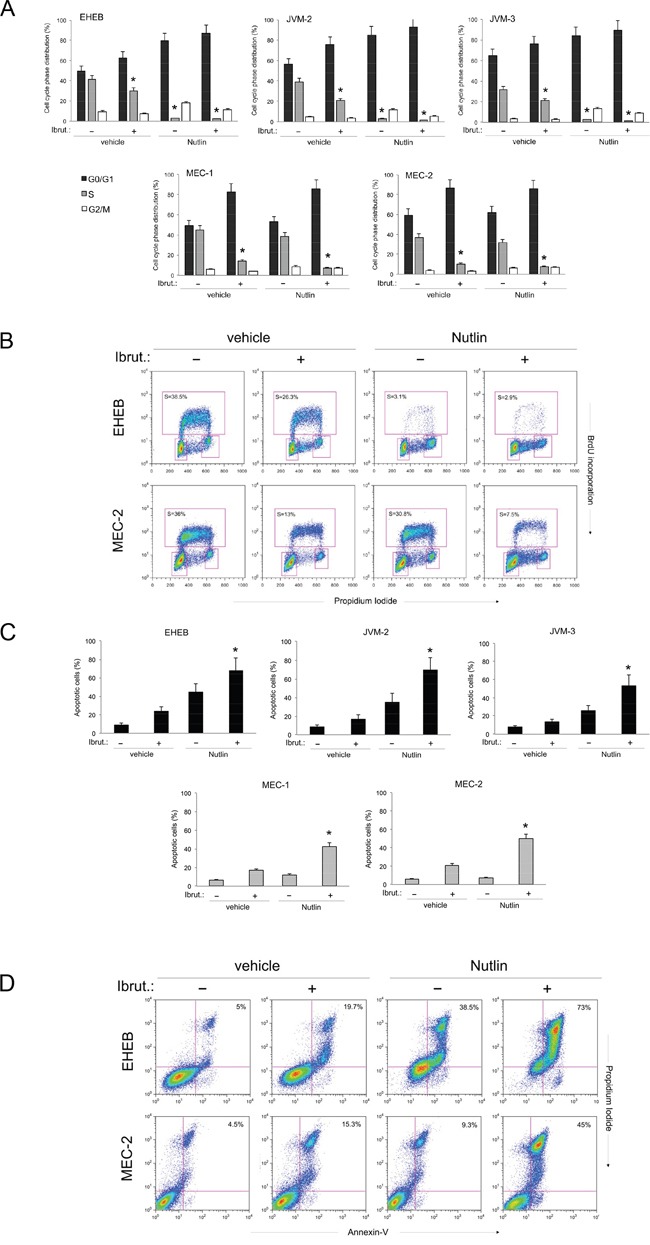
Effects of Ibrutinib/Nutlin-3 combination on cell cycle and apoptosis in B leukemic cell lines The p53^wild-type^ (EHEB, JVM-2, JVM-3) and the p53^mutated^ (MEC-1, MEC-2) leukemic cells were exposed to Ibrutinib (10 μM) and Nutlin-3 (10 μM) used either alone or in combination, before flow cytometric analysis of cell cycle progression A-B and apoptosis induction C-D, evaluated after 24 and 48 hours, respectively. In **A.** cell distribution in the different phases of the cell cycle was calculated from the flow cytometric dot plots after BrdU/PI staining and is expressed as percentage of the total population. The asterisk indicates p<0.05 respect to control vehicle. In **B.** representative flow cytometric dot plots of cell-cycle profiles were analyzed by BrdU incorporation after the indicated treatments. The rectangles represent the cells in the different (G0/G1, S, G2/M) phases of the cell cycle and the percentage of cells in S-phase is indicated for each treatment. In **C.** induction of apoptosis was calculated as percentage of Annexin V/PI double positive cells on the total population for each treatment. The asterisk indicates p<0.05 respect to single treatments. In **D.** representative plots of apoptotic cells were analyzed by flow-cytometry following Annexin-V/PI staining. The percentage of apoptotic cells is indicated for each treatment. In A and C, data are reported as the mean±SD of results from at least three independent experiments.

Based on the cell viability data, we have next investigated the effect of Ibrutinib/Nutlin-3 combination both on the cell cycle progression (Figure 2A-2B) as well as on apoptosis modulation (Figure 2C-2D). As reported in Figure 2A, treatment with Ibrutinib alone induced a significant reduction of S phase accompanied by a concomitant increase in G0/G1 phase in all B lymphoblastoid cell lines. In the p53^wild-type^ leukemic cell lines, the combination of Ibrutinib/Nutlin-3 further enhanced the accumulation of cells in G0/G1 phase due to the marked cytostatic activity of Nutlin-3 in these cells (Figure 2A-2B). On the other hand, the cell cycle profile of p53^mutated^ B leukemic cell lines was very similar in cells treated with Ibrutinib alone or Ibrutinib/Nutlin-3 combination since Nutlin-3 was not effective in these cells (Figure 2A-2B). Therefore, both in the p53^wild-type^ and p53^mutated^ leukemic cell lines, the effect of the drug combination on cell cycle was masked by the predominant effect of each single drug. At the opposite, the analysis of apoptosis showed that Ibrutinib alone induced modest levels of apoptosis in all cell lines, while the Ibrutinib/Nutlin-3 combination significantly increased the percentage of apoptosis with respect to the treatment with the single drugs used alone, in both p53^wild-type^ and p53^mutated^ B leukemic cell lines (Figure 2C-2D). These data clearly suggest that the induction of apoptosis, rather than the cell cycle block mainly accounted for the synergistic anti-leukemic activity of the Ibrutinib/Nutlin-3 combination.

**Figure 3:**
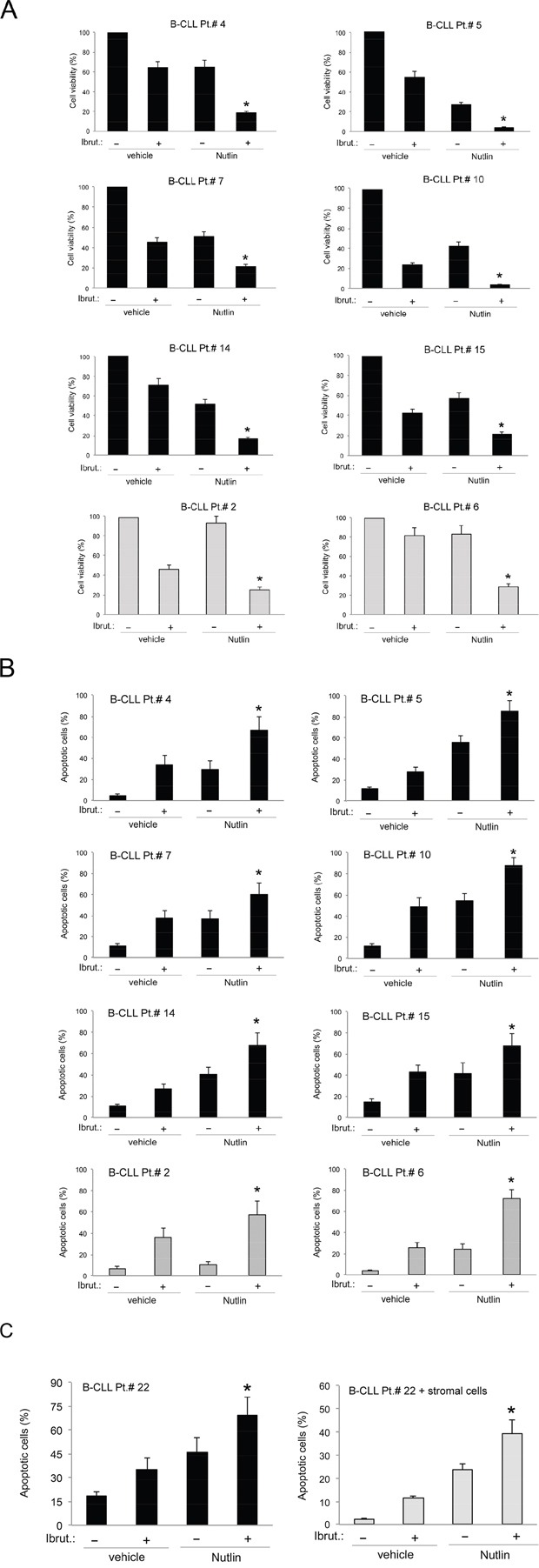
Effects of Ibrutinib/Nutlin-3 combination in primary B-CLL patient cell cultures B-CLL patient (Pt.) samples were exposed to Ibrutinib (10 μM) and Nutlin-3 (10 μM) used either alone or in combination for 24-48 hours. In **A.** cell viability in response to each treatment was calculated as percentage with respect to the control untreated cultures (set to 100% for each primary sample). In **B.** induction of apoptosis was calculated as percentage of Annexin V/PI double positive cells on the total population. In A and B, p53^wild-type^ (black) and p53^mutated^ (gray) B-CLL patient samples are shown. In **C.** induction of apoptosis was calculated as percentage of Annexin V/PI double positive cells on the total population of cells treated either in suspension or in presence of a monolayer of stromal cells mimicking the microenvironment. In A, B and C, data are reported as the mean±SD of results from at least three independent experiments. Representative primary B chronic lymphocytic leukemia samples are shown. The asterisk indicates p<0.05 respect to single treatments.

To ascertain the potential clinical relevance of the data obtained in B leukemic cell lines, we have investigated the effect of Ibrutinib/Nutlin-3 combination in experiments performed in primary B-CLL samples. As for B leukemic cell lines, treatment of primary B-CLL cells with serial concentrations of Ibrutinib and Nutlin-3 at a constant ratio revealed a synergistic (average CI<1) cytotoxic activity in 10 out of the 11 primary B-CLL samples analyzed (Table 4). The synergistic effect of the Ibrutinib/Nutlin-3 combination was independent from the p53 status of the patient's cells, confirming the data obtained in B-cell lines. Moreover, also in primary B-CLL cells the cytotoxicity induced by the Ibrutinib/Nutlin-3 combination was mainly due to the increase of apoptosis with respect to the treatment with either Ibrutinib or Nutlin-3, used alone (Figure 3A-3B). As a matter of fact, effects on cell cycle were not considered because B-CLL primary cells derived from peripheral blood and cultured *ex vivo* are mostly in G0/G1 phase and quiescent not replicating. In light of the well-known pro-survival role of the microenvironment on leukemic cells [23], we have then assessed the effect of Ibrutinib/Nutlin-3 combination on B-CLL cells co-cultured on a monolayer of stromal cells (mimicking the disease microenvironment). Despite the protective effect resulting in general reduction of apoptosis levels respect to culture of B-CLL cells alone, the combined treatment still exhibited a synergistic effect in apoptosis induction (Figure 3C).

**Table 4:** Combination index values for the effect of Ibrutinib/Nutlin-3 combination on viability of B-CLL patient cells

Leukemic cells	ED50	ED75	ED90	Average CI
Patient #1	0.91	0.67	0.50	0.69
Patient #2	0.50	0.54	0.58	0.54
Patient #3	1.02	0.39	0.30	0.57
Patient #7	0.37	0.20	0.11	0.23
Patient #9	1.19	1.43	1.76	1.46
Patient #10	0.34	0.21	0.13	0.23
Patient #11	0.74	0.37	0.19	0.43
Patient #12	0.28	0.20	0.18	0.22
Patient #14	0.16	0.08	0.04	0.09
Patient #15	0.52	0.21	0.10	0.28
Patient #16	0.56	0.33	0.20	0.36

ED indicates effect dose. The average combination index (CI) values were calculated from ED50, ED75 and ED90.

A potential pitfall of our study is represented by the fact that Nutlin-3 showed poor *in vivo* bioavailability [24], a finding that hampered its potential clinical use. In this regard, a novel MDM-2 inhibitor (RG7112) with superior bioavailability with respect to Nutlin-3 is currently under evaluation in clinical trials in patients with hematologic malignancies [25]. Of note, similarly to Ibrutinib/Nutlin-3 combination, also Ibrutinib/RG7112 combination promoted synergistic cytotoxicity of B leukemic cells (Supplementary Figure S1), having average CI values <1 for both p53^wild-type^ EHEB (0.82 and 0.21 at 24 and 48 hours of treatment, respectively) as well as p53^mutated^ MEC-2 cells (0.99 and 0.59 at 24 and 48 hours of treatment, respectively).

### Intracellular mechanisms responsible for the anti-leukemic effect of Ibrutinib/Nutlin-3 combination

**Figure 4:**
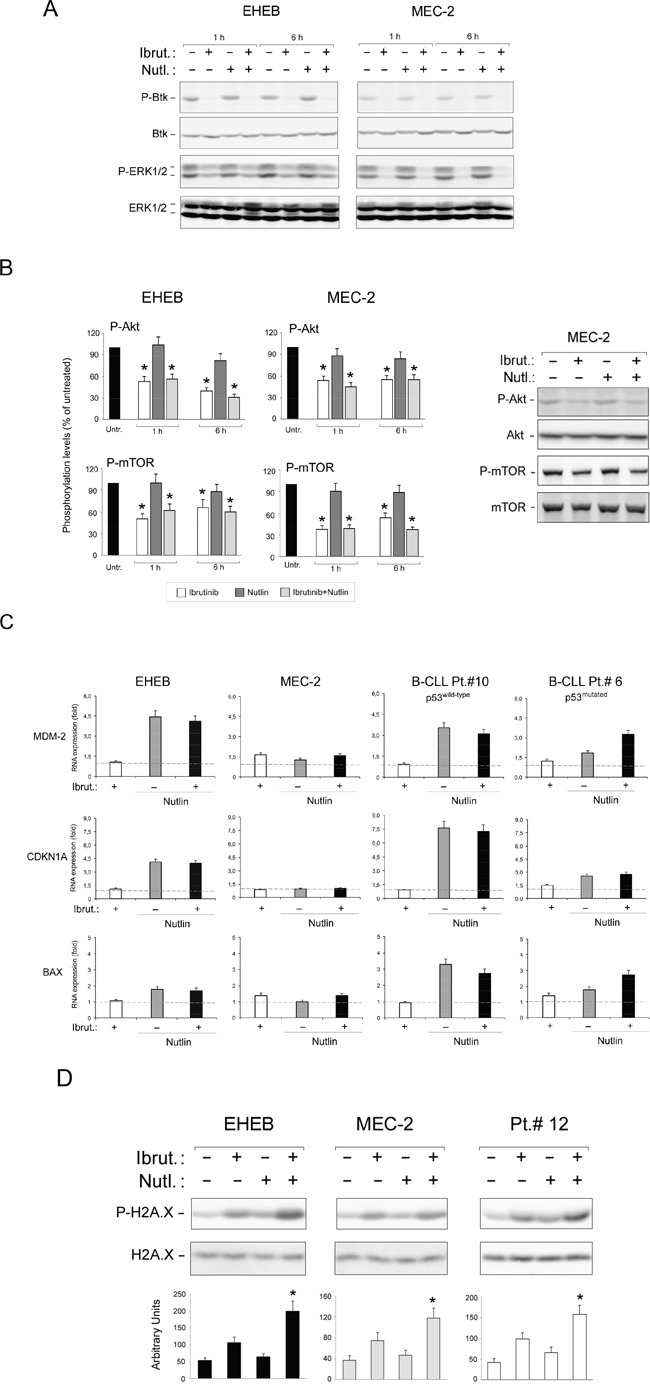
Intracellular pathway mediating the anti-leukemic activity of Ibrutinib/Nutlin-3 combination EHEB (p53^wild-type^) and MEC-2 (p53^mutated^) leukemic cell lines, as well as representative B-CLL patient (Pt.) samples, were exposed to Ibrutinib (10 μM) and Nutlin-3 (10 μM) used either alone or in combination and were evaluated at different time points, as indicated. In **A.** western blotting analysis of activated (phosphorylated) and total Btk and ERK1/2 protein levels are shown. In **B.** phosphoprotein expression levels for Akt and m-TOR were measured by fluo-immunobeads assays. Analyses of phosphoprotein levels were performed by Luminex system and results are indicated as folds of induction with respect to the control untreated cultures set at 100. The asterisk indicates p<0.05 respect to the untreated. Validation by western blotting analysis is shown. In **C.** levels of *CDKN1A*, *MDM2* and *BAX* were analyzed after 24 hours of treatment by quantitative RT-PCR. mRNA levels are expressed as fold of modulation with respect to the control untreated cultures set at 1. Results are reported as means±SD of at least three independent experiments, performed in duplicate. The asterisk indicates p<0.05 respect to control vehicle. In **D.** western blotting analysis of activated and total H2A.X protein levels performed on B-leukemic cell lines and primary B-CLL samples. The asterisk indicates p<0.05 respect to single treatments. In A, B and D, blots representative of at least three independent experiments yielding equivalent results are shown.

It is well known that the pathogenesis of B-CLL is characterized by alterations of cellular signaling pathways. Therefore, in the next group of experiments, we have evaluated if the cellular responses observed after treatment with Ibrutinib/Nutlin-3 combination correlated with modification of intracellular signaling events, with particular attention to pathways regulated by BTK and/or p53 that are targets of Ibrutinib and Nutlin-3, respectively (Figure 4). In line with previous studies [26, 27], by western blotting analyses we found that exposure of B leukemic cell cultures to Ibrutinib resulted in inhibition of BTK auto-phosphorylation at tyrosine 223 (caused by the inhibition of kinase activity by the binding of Ibrutinib) coupled to a significant reduction of the phosphorylation of the MAPK survival pathway modulators ERK1/2, starting at early time points post drug exposure (Figure 4A). Moreover, by assessing phosphorylation levels through magnetic-plex assays, we observed a rapid down-modulation of the PI3K survival pathway, as documented by the reduction of both Akt and m-TOR phosphorylation, which was validated also by western blotting (Figure 4B). Independently by the p53 status of the tested cells, superimposable results were observed also after treatment with Ibrutinib/Nutlin-3 combination, indicating that Nutlin-3 treatment did not significantly affect these pathways (Figure 4A-4B). On the other hand, while Nutlin-3 treatment induced activation of the p53 pathway in p53^wild-type^ B-leukemic cells, as documented by the significant increase of the expression levels of *CDKN1A*, *MDM2* and *BAX* genes, no effects on this pathway were observed upon treatment with Ibrutinib used either alone or in combination (Figure 4C). No significant modulation of the p53 pathway was observed in p53^mutated^ B cells following any treatment (Figure 4C).

Finally, considering the emerging role of the histone protein H2A.X for the clinical validation of anticancer candidate drugs [28, 29], we have then evaluated if the anti-leukemic activity in our setting was associated with DNA damage response (DDR). Western blotting analysis on cellular lysates from B-leukemic cell lines highlighted that the exposure to Ibrutinib was associated to up-regulation of phospho-H2A.X, responsible for the DDR-signal amplification, and that this response was further enhanced by the combination with Nutlin-3 (Figure 4D). Of note, the activation of DDR at early time points, before the onset of the apoptosis, was observed both on leukemic cell lines (independently from the p53 status) as well as on primary cells derived from B-CLL patients (Figure 4D).

### The Ibrutinib/Nutlin-3 combination promotes survival and is associated to induction of phospho-H2A.X in mouse tumor tissues

**Figure 5:**
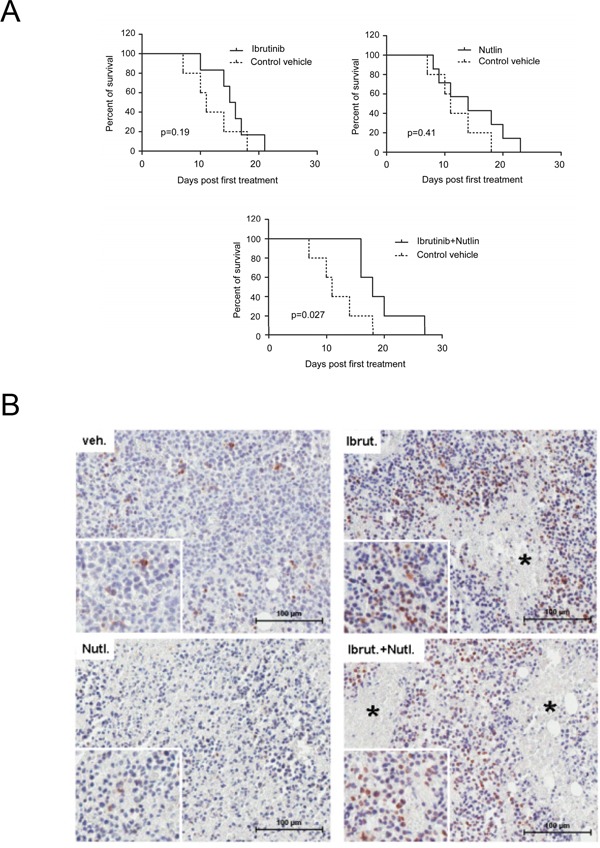
Anti-leukemic activity of Ibrutinib/Nutlin-3 combination assessed *in vivo* in a xenograft murine model SCID mice were subcutaneously injected with JVM-2 cells (10^7^ cells/mouse). When xenograft tumors reached 50 mm^3^ of volume, mice were locally injected with control vehicle, Nutlin-3, Ibrutinib or Nutlin-3/Ibrutinib combination. In **A.** survival is reported as percentage measured from the day of the first treatment. In **B.** immunohistochemical analysis was performed with antibody anti-phospho H2A.X on sections of subcutaneous masses. Magnification 10X and 40X (inserts). Asterisks show areas of necrosis.

To preliminarly validate the *in vitro* results in an *in vivo* model, we adopted a JVM-2 xenograft subcutaneous model generated in SCID mice. When tumors reached 50 mm^3^, JVM-2 xenograft mice were treated s.c. with control vehicle, Ibrutinib, Nutlin-3 or Ibrutinib/Nutlin-3 combination. Only the combination treatment was associated to significant (p<0.05) increase in survival as compared to control xenografts (Figure 5A). Moreover, immunohistochemistry analysis for phospho-H2A.X performed on the tumoral mass of sacrificed mice showed low background in mice inoculated with either vehicle or Nutlin-3, and a strong phosphorylation of H2A.X protein in the nucleus of cells forming the tumoral tissue of mice treated with Ibrutinib (used either alone and in combination with Nutlin-3), with higher signals localized in proximity of areas of necrosis (Figure 5B).

## DISCUSSION

In B-CLL, the FCR regimen (fludarabine, cyclophosphamide, rituximab) continues to represent the ‘standard of care’, and an existing weight of evidence demonstrates a survival advantage for FCR over historical approaches, at least for younger patients (<65 years) without *TP53* aberrations [30]. Nevertheless, Ibrutinib represents currently a substantial therapeutic advance in B-CLL [7]. Several ongoing clinical trials are evaluating Ibrutinib broadly as first-line treatment, alone or in combination with anti-CD20 monoclonal antibodies, as compared with chemo-immunotherapy regimens (FCR, bendamustine-rituximab, obinutuzumab-chlorambucil) [31, 32]. However, since resistances to Ibrutinib therapy are emerging [10–13], innovative combinations of Ibrutinib with small molecules that block adaptive signaling responses are starting to be investigated in the preclinical setting [33, 34].

In this context, we have explored the effects of the combination of Ibrutinib with the small molecule Nutlin-3, based on the rationale that in clinical approaches Ibrutinib would “mobilize” B-CLL cells from their protective microenvironment [35], and together with Nutlin-3 would target them in the circulation where they are more susceptible to apoptotic stimuli. Our *in vitro* studies on leukemic cell models and primary cells from B-CLL patients have confirmed this hypothesis, showing a synergistic cytotoxic effect of Ibrutinib/Nutlin-3 combination in both p53^wild-type^ and p53^mutated^ cells. In addition, a synergistic mechanism of action, thought apoptosis induction, was also documented on B-CLL cells co-cultured with cells mimicking the tumor microenvironment [36], further strengthening the potential therapeutic significance of our current data.

With respect to the molecular mechanism underlying the Ibrutinib/Nutlin-3 combination, it has been clearly established that an activated B-cell receptor signaling pathway and a disturbed DNA damage response (DDR) play a major role in promoting B-CLL cell survival [22]. External stimuli that lead to activation of the MAPK and PI3K/AKT pathways are similarly essential for B-CLL cell survival [23]. Therefore, while confirming that Ibrutinib (either alone or in combination with Nutlin-3) marked counteracts the MAPK and PI3K/AKT pathways in B-CLL, we have provided evidence that the synergistic anti-leukemic activity of the Ibrutinib/Nutlin-3 combination have a convergence point in regulating cell survival/death through the activation of the DDR signaling. This observation was documented *in vitro*, in both cell lines as well as in patient cells cultures, and was preliminarily confirmed in mice xenograft, where the Ibrutinib/Nutlin-3 combination induced a survival advantage over the single treatments and activation of the H2A.X histone protein in the tumoral tissues. Certainly, additional experiments, performed on NSG xenotransplant B-CLL mice generated using patient cells [37], will be needed to further clarify this point in *in vivo* models. Interestingly, activation of H2A.X has recently been involved also in mediating the anti-tumoral activity of 5-fluorouracil-based combinations in a model of colon cancer [38].

Overall, our data suggest that the Ibrutinib/MDM-2 inhibitor combination merits further investigation for its therapeutic potential. The first non-genotoxic specific small-molecule antagonist of the MDM-2-p53 binding interaction, Nutlin-3, has been used extensively as a probe compound in preclinical and mechanistic studies, but it did not enter into clinical use due to its poor *in vivo* bioavailability [24]. Anyhow, the second generation MDM-2 inhibitors with superior potency and oral bioavailability, such as RG7112 [25], will enter into clinics. In particular, RG7112 showed promising therapeutic activity in phase I clinical trial in hematological malignancies, including B-CLL [25]. Considering these evidences and the fact that not only the p53 wild-type but also some p53 mutated patients of the clinical trial responded to RG7112 [25, 39], it is noteworthy that also RG7112 synergizes with Ibrutinib in promoting cytotoxicity of B leukemic cells. This preliminary data is indeed encouraging for the advance of the drug combination towards the clinic.

## MATERIALS AND METHODS

### B leukemic cell lines and primary B-CLL patient samples

The B leukemic cell lines EHEB, JVM-2 and JVM-3 (p53^wild-type^) as well as MEC-1 and MEC-2 (p53^mutated^) were purchased from DSMZ (Deutsche Sammlung von Mikroorganismen und Zellkulturen GmbH, Braunschweig, Germany). EHEB, JVM-2 and JVM-3 were routinely cultured in RPMI-1640, while MEC-1 and MEC-2 were maintained in IMDM, both media supplemented with 10% FBS, L-glutamine and penicillin/streptomycin (all from Gibco, Grand Island, NY) [40].

For experiments with primary cells, peripheral blood samples were collected in heparin-coated tubes from B-CLL patients, and from healthy blood donors used as controls, following informed consent, in accordance with the Declaration of Helsinki and in agreement with institutional guidelines (University-Hospital of Ferrara). The diagnosis of B-CLL was made by peripheral blood morphology and immunophenotyping. The main clinical parameters of the B-CLL patients were abstracted from clinical records and all patients had been without prior therapy at least for three weeks before blood collection. Peripheral blood mononuclear cells (PBMC) were isolated by gradient centrifugation with lymphocyte cell separation medium (Cedarlane Laboratories, Hornby, ON). T lymphocytes, NK lymphocytes, granulocytes and monocytes were negatively depleted from B-CLL PBMC with immunomagnetic microbeads (MACS microbeads, Miltenyi Biotech, Auburn, CA), with a purity >95% of resulting CD19+ population. Freshly isolated and thawed primary cells were cultured in RPMI-1640 medium containing 10% FBS, L-glutamine and penicillin/streptomycin (Gibco), as previously described [41].

### Culture treatments and assessment of cell viability, cell cycle profile and apoptosis

For *in vitro* treatments with Ibrutinib (PCI-32765) (Selleckchem, Houston, TX), used either alone or in combination with Nutlin-3 (Cayman Chemicals, Ann Arbor, MI), cells were seeded at a density of 1x10^6^ cells/mL. In selected experiments, Ibrutinib was assessed also in combination with the RG7112 MDM2 inhibitor (Selleckchem). At different time points after treatment, cell viability was examined by Trypan blue dye exclusion and MTT (3-(4, 5-dimethilthiazol-2yl)-2, 5-diphenyl tetrazolium bromide) colorimetric assay (Roche Diagnostics Corporation, Indianapolis, IN) for data confirmation, as previously described [41, 42]. In order to investigate the concentration required to induce death in 50% of cells respect to control, IC_50_ values were calculated from dose-response curves constructed by plotting cell survival (%) versus drug concentration. The cell cycle profile was analyzed by 5-bromodeoxyuridine (BrdU) incorporation assessed by flow cytometry, as previously described [43]. Levels of apoptosis were quantified by Annexin V-FITC/propidium iodide (PI) staining (Immunotech). To avoid non-specific fluorescence from dead cells, live cells were gated tightly using forward and side scatter, as described [44].

### Protein analyses

For western blotting analysis, cells were lysed as previously described [45]. Protein determination was performed by BCA Protein Assay (Thermo Scientific, Rockford, IL). Equal amounts of protein for each sample were migrated in SDS-polyacrylamide gels and blotted onto nitrocellulose filters. The following Abs were used: anti-Btk (C82B8), anti-phospho-Btk (Tyr223), anti-mTOR, anti-phospho-mTOR (Ser2448), anti-histone H2A.X and anti-phospho-histone H2A.X (Ser139) all from Cell Signaling (Danvers, MA); anti AKT/PKBα from Becton-Dickinson; anti-phospho-Akt1/PKBα (Ser473) from Merck Millipore (Darmstadt, Germany); anti-p44/42 MAPK (ERK1/2) and anti-phospho-Thr202/Tyr204 ERK1/2 from Promega (Madison, USA). After incubation with anti-mouse or anti-rabbit IgG horseradish peroxidase-conjugated secondary antibodies (Sigma-Aldrich), specific reactions were revealed with the ECL Lightning detection kit (Perkin Elmer, Waltham, MA) [46]. Images acquisition was performed using the ImageQuant™ LAS 4000 biomolecular imager (GE Healthcare, Buckinghamshire, UK) and densitometry values were estimated by the ImageQuant TL software (GE Healthcare).

In selected experiments, cell lysates were analyzed for the detection of phosphoproteins and relative total target proteins by using fluorescently died magnetic bead-based immunoassays (Bio-Plex Pro Phosphoprotein magnetic 8-plex and Total Target magnetic 7-plex, BioRad Laboratories, Hercules, CA), accordingly to the manufacturer's instructions. Data were acquired using a MAGPIX® system (Luminex, Austin, TX), analyzed with the xPONENT® software (Luminex) and reported as Median Fluorescence Intensity (MFI).

### RNA analyses

Total RNA was extracted from cells using the QIAGEN RNeasy Plus mini kit (QIAGEN, Hilden, Germany), accordingly to the supplier's instructions. Total RNA was transcribed into cDNA and amplified using the Express One-Step Superscript qRT-PCR Kit (Invitrogen, Carlsbad, CA). Analysis of human *CDKN1A*, *MDM2* and *BAX* gene expression was carried out with validated TaqMan Gene Expression Assays specific PCR primers sets (Invitrogen). All samples were run in triplicate using the real time thermal analyzer Rotor-Gene™ 6000 (Corbett, Cambridge, UK), as previously described [47]. Expression values were normalized to the housekeeping gene *POLR2A* amplified in the same sample.

### Experiments in mouse models

Female cb17/SCID mice (5 weeks-old) were purchased from Charles River Laboratories (Hollister, CA) and maintained in accordance with the guide for the care and use of laboratory animals at the animal facility of the University of Ferrara. Mice were housed in vented cabinet with food and water ad libitum. The procedures involving animals and their care were approved by the institutional animal ethical care committee of the University of Ferrara (OBA) and by the Italian Ministry of Health. JVM-2 (10^7^) B leukemic cells were harvested, washed and suspended in PBS before subcutaneous injection (in a volume of 100 μL) into the right dorsum of 6-week-old mice, as previously described [48]. Tumor growth was determined by caliper measurements of two orthogonal axes and the tumor volume was calculated by the formula: *L×l*^2^×*0.5*, wherein *l* is the shorter and *L* is the longer axis; the tumor density was assumed to be equal to one. When tumors reached 50 mm^3^ of volume, leukemia xenograft mice were randomized into groups (of at least 8 mice each) receiving every other day for a total of five times subcutaneous intra-tumoral injections (in 100 μL PBS/30% DMSO) of Ibrutinib (2.2 mg/Kg), Nutlin-3 (2.9 mg/Kg) or Ibrutinib/Nutlin-3 combination (2.2 and 2.9 mg/Kg, respectively). Control group was represented by mice injected with vehicle (PBS/30% DMSO). Animals were monitored daily for changes in weight, side effects or signs of sickness. Survival was calculated as the duration of the animal life span from the inoculation of first treatment until sacrifice when excessive signs of sickness were observed. For histological analysis, the subcutaneous masses were fixed in 10% buffered-formalin solution and embedded in paraffin. Five-μm-thick sections were cut from paraffin blocks and stained with hematoxylin-eosin and/or used for immunohistochemistry with the Ab anti-phospho-histone H2A.X (Ser139) (Cell Signaling) and the anti-rabbit HRP-DAB tissue staining kit (R&D System, Minneapolis, MN). In each slide, a negative control was obtained carrying out the immunohistochemistry procedure without the primary antibody. Sections were acquired with an Aperio ScanScope® slide scanner by using the Aperio ImageScope v11.1.2.760 software (Leica Biosystems, Nussloch, Germany).

### Statistical analysis

Results were evaluated by using analysis of variance with subsequent comparisons by Student's t-test and with the Mann-Whitney rank-sum test. Statistical significance was defined as p<0.05. In order to investigate the effect of drug combinations, leukemic cells were treated with serial doses (range 1-10 μM) of each drug, used individually or in combination, using a constant 1:1 ratio. Results were analyzed with the method of Chou and Talalay [22] to determine whether the combined treatment yields greater effect than expected from summation: a combination index (CI) of 1 indicates additive effect, while a CI below 1 indicates synergism. For this purpose, cell viability datawere analyzed with the CalcuSyn software and reported as CI values.

For the experiments in mice, analysis of survival data was carried out with GraphPad Prism version 5 (GraphPad Software). In particular differences in survival between treatment groups were calculated using the Kaplan-Meier curve and survival distribution of the treated and control groups was compared using the Gehan-Breslow-Wilcoxon test. Differences were considered significant when *p* value was <0.05.

## SUPPLEMENTARY FIGURE


