# Supplementary material for: Ibrutinib synergizes with MDM-2 inhibitors in promoting cytotoxicity in B chronic lymphocytic leukemia
Source: Oncotarget. 2016 Sep 20;7(43):70623–38. doi: 10.18632/oncotarget.12139 (PMC5342579; doi:10.18632/oncotarget.12139)
Supplement: Supplementary file 1 [file oncotarget-07-70623-s001.pdf]

Ibrutinib synergizes with MDM-2 inhibitors in promoting cytotoxicity in B chronic lymphocytic leukemia

SUPPLEMENTARY FIGURE

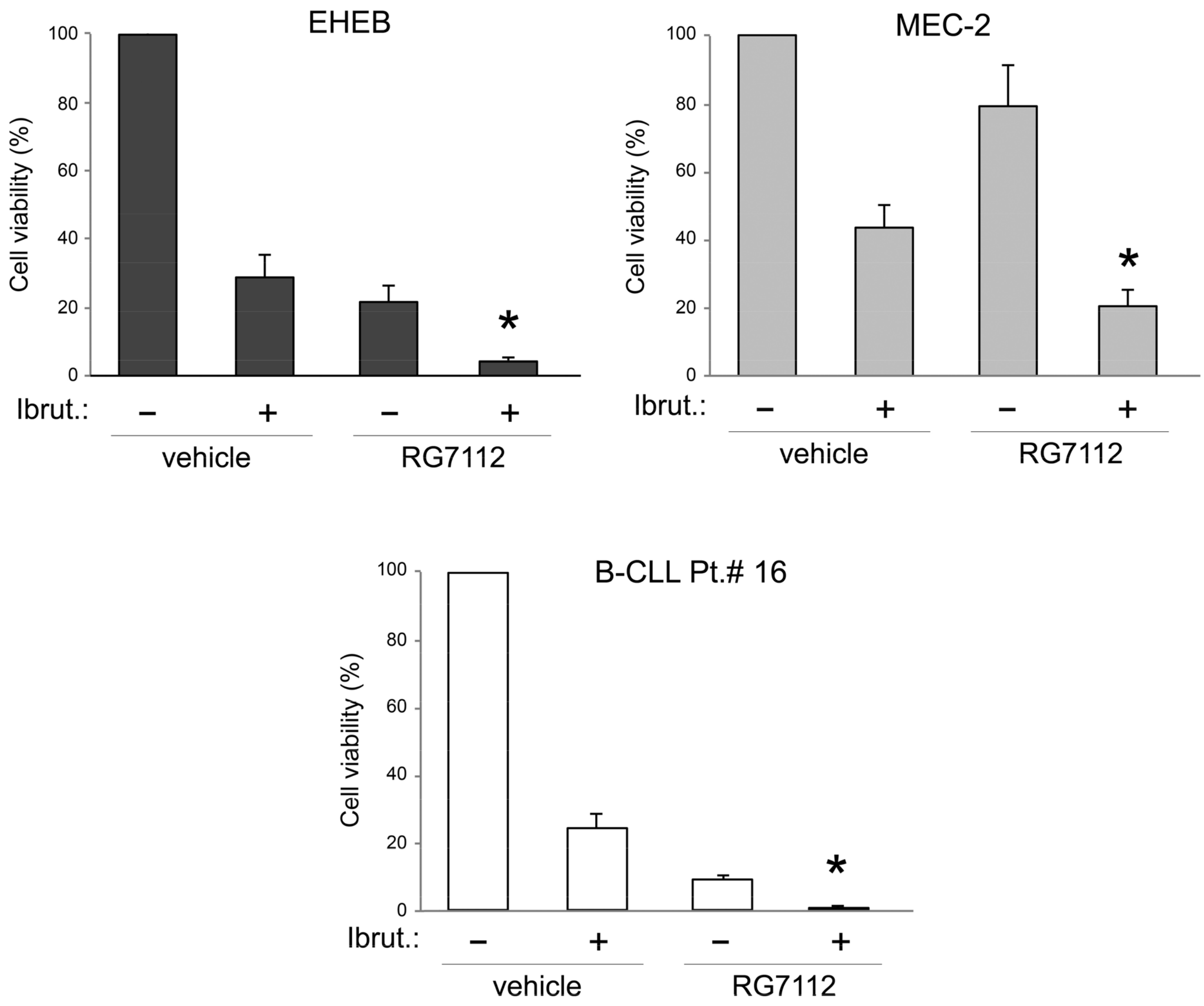

Supplementary Figure S1: Evaluation of cytotoxic effect in response to Ibrutinib/RG7112 combination in B leukemic cell lines. The p53[wild-type] EHEB and the p53[mutated] MEC-2 leukemic cells as well as cells of a representative B-CLL patient (Pt.) sample were exposed to Ibrutinib (10 µM) and RG7112 (10 µM) used either alone or in combination for 48 hours. Cell viability was calculated as percentage with respect to the control untreated cultures (set to 100% for each cell culture). Data are reported as the mean±SD of results from at least three independent experiments. The asterisk indicates $p<0.05$ respect to single treatments.
